# Reactive oxygen species turnover, phenolics metabolism, and some key gene expressions modulate postharvest physiological deterioration in cassava tubers

**DOI:** 10.3389/fmicb.2023.1148464

**Published:** 2023-02-28

**Authors:** Elizabeth Devi Wahengbam, Chingakham Premabati Devi, Susheel Kumar Sharma, Subhra Saikat Roy, Albert Maibam, Madhumita Dasgupta, Star Luikham, Tania Chongtham, Arati Ningombam, Ingudam Bhupenchandra, Laishram Kanta Singh, Yumnam Prabhabati Devi, Sushmita Thokchom, Chingakham Inao Khaba, Nameirakpam Bunindro Singh, Yallappa Rajashekar, Sudripta Das, Sansuta Mohanty, Manas Ranjan Sahoo

**Affiliations:** ^1^ICAR Research Complex for North Eastern Hill Region, Imphal, Manipur, India; ^2^Farm Science Centre, ICAR Research Complex for North Eastern Hill Region, Imphal, Manipur, India; ^3^Assam Agricultural University, Jorhat, Assam, India; ^4^Institute of Bioresources and Sustainable Development, Imphal, Manipur, India; ^5^Central Horticultural Experiment Station, ICAR–Indian Institute of Horticultural Research, Bhubaneswar, Odisha, India

**Keywords:** cassava, metabolites, phenolics, postharvest physiological deterioration, reactive oxygen species, signaling genes

## Abstract

Rapid postharvest physiological deterioration (PPD) in cassava (*Manihot esculenta* Crantz) tuber is a significant concern during storage. The freshly harvested tubers start spoiling within 24 to 72 h. Accumulation of H_2_O_2_ is one of the earliest biochemical events that occurred during PPD, which was detected using the 3,3 diaminobenzidine (DAB) in two contrast cassava genotypes, MNP Local A (29–57 μg g^–1^) and Sree Prakash (64–141 μg g^–1^). Accumulating the fluorescence hydroxycoumarin compounds emitted by the cassava tubers observed under an ultraviolet (UV) lamp showed significant variations at 0, 3, 6, 9, 12, and 15 days of storage. The total phenolics and carotenoids significantly and negatively correlated with PPD progression; however, the anthocyanin and flavonoids positively correlated with the PPD–anchored ROS accumulation. The primary compound, Phthalic acid, di(2–propylpentyl) ester, was identified in both the cassava tubers, Sree Prakash (57.21 and 35.21%), and MNP Local A (75.58 and 60.21%) at 0, and 72 h of PPD, respectively. The expression of PPD–associated genes *APX–2, APX–3, PAL*, and *AP* was higher at 6–12 days of PPD, which signified the synthesis of ROS turnover and phenylpropanoid biosynthesis. A significant, strong, and positive correlation was established between the secondary metabolites and PPD signaling gene expression, which was inversely correlated with hydroxycoumarin and H_2_O_2_ accumulation. MNP Local A tubers exhibited longer storage life of 15 days with a low PPD score, higher metabolites synthesis, and gene expression. The PPD–resistant lines may be used to augment cassava breeding strategies for large–scale commercial and industrial use.

## 1. Introduction

Cassava (*Manihot esculenta* Crantz), which belongs to the Euphorbiaceae family, is one of the important tuber crops grown in the tropics of the world for energy sources (Devi and Singh, 2017). It is also called manioc or tapioca, consumed as the most important staple root and tuber crop worldwide. Cassava ranks sixth after maize, rice, wheat, potatoes, and soybeans (Zainuddin et al., 2018), with a global production of 304 million tons in FAOSTAT (2021). Initially, cassava was considered a crop for resource–poor farmers and designated as an Orphan crop (Azadi et al., 2016), but its value has gained over time. Now cassava is one of the most widely cultivated root crops and constitutes the source of food supplements and provides the food energy intake for over one billion people worldwide. In developing countries, cassava is considered a crop for sustainable agriculture and food security. It can grow well in infertile soils, having drought tolerance and low susceptibility to pathogens. It has a high carbohydrate yield and is the cheapest starch source in more than 300 industrial products (Zainuddin et al., 2018). In the northeastern hill region of India, cassava is well adopted by more than 166 tribal communities for food and animal feed with high–income generation. At the time of food scarcity in this region, the tuber crops, cassava in particular only supply dietary energy for survival (Devi and Singh, 2017).

Despite the potential accomplishment, cassava production remains inhibited by many biotic and abiotic factors (Campo et al., 2011). One of the significant constraints is that cassava has less shelf life and is vulnerable to postharvest physiological deterioration (PPD). The cassava roots exhibit physiological deterioration within only 24 to 72 h of harvest (Vanderschuren et al., 2014). Due to this, the tubers became non–edible and lost their marketability, severely limiting their benefits to cassava growers (Zainuddin et al., 2018).

Postharvest physiological deterioration occurs in two stages of deterioration processes: physiological or primary deterioration and secondary microbiological deterioration. Initially, in primary deterioration, a visible sign of blackish–blue to black discoloration appears in the vascular tissue of the root, which begins at the cut or broken surfaces. After that, the discoloration outspread to the adjacent storage parenchyma, and the stored starch undergoes structural changes causing more discoloration and unsatisfactory cooking qualities. Primary deterioration is a complex physiological and biochemical process that occurs due to mechanical injury during harvest and further exposure to oxygen, resulting in the overproduction of reactive oxygen species (ROS), such as H_2_O_2_, leading to cellular dysfunction. On the other hand, plants deployed secondary metabolites (phenolic, anthocyanin, flavonoids, carotenoids, etc.) to impair the oxidative burst (Zainuddin et al., 2018). Antioxidative enzymes play a crucial role in equilibrating the oxidative burst and normalizing cellular function through various pathways (Uarrota et al., 2014).

Postharvest physiological deterioration in cassava tubers is a complex physiological and biochemical process that involves ROS as an early event occurred due to the oxidation of cellular components (Reilly et al., 2004). ROS turnover primarily fine–tunes the PPD syndrome. Thus, understanding the mechanisms of ROS overproduction would be essential to predicting a crosstalk model on the ROS scavenging mechanism (Djabou et al., 2017). The occurrence of ROS under PPD progression could be estimated calorimetrically and localized using 3,3 diaminobenzidine (DAB) as dark brown precipitation in the presence of haem–containing protein. Furthermore, PPD resulted in vascular streaking, xylem strands, and parenchyma discoloration due to the rapid accumulation of a fluorescence compound, hydroxycoumarin (Reilly et al., 2007). Ultraviolet (UV) visible fluorescence hydroxycoumarin accumulated in the cassava tubers upon PPD progression, which was low in fresh tubers.

On the other hand, synthesizing metabolites, such as phenolic accumulation, is an asset to the cellular component that eliminates the negative consequences of ROS–anchored PPD. H_2_O_2_ and phenolics are often used as biomarkers for assessing cassava PPD (Zainuddin et al., 2018). Phenolics are the crucial secondary metabolites that constitute the phenylpropanoids group with aromatic rings of one or more OH^–^ groups (Albuquerque et al., 2021). OH^–^ groups further participate in the chemical bonding with H_2_O_2_ to form singlet O_2_. Flavonoids and anthocyanins are the natural bioactive compounds in the phenolic group that contain similar aromatic rings and play a potent ROS inhibitor. Similarly, carotenoids are tetraterpenoids–based natural antioxidants that contribute to total antioxidant capacity and deactivate cellular free radicals (Safafar et al., 2015).

Several genes associated with ROS turnover and phenolics biosynthesis rapidly upregulated upon progressive PPD, which substantiates the active and degenerative role of PPD in cassava (Zainuddin et al., 2018). Genes of ascorbate peroxidase, *APX* family, played a crucial role in ROS turnover as an early event at 3–6 days of PPD. Phenylalanine ammonia–lyase (*PAL*) regulates phenylpropanoid biosynthesis in cassava tubers during PPD (Vanderschuren et al., 2014). Aspartic protease (*AP*), a wound–response gene associated with PPD in cassava, regulates ROS production and phenolic compounds (Fernando et al., 2002). PPD–associated gene regulation confirms the mechanisms of visual scoring, biochemical events, and metabolite synthesis (Reilly et al., 2007).

Different methodologies were adopted for PPD scoring, which involves visual scales using entire root slices, the visual scoring method in part of the root slices (Saravanan et al., 2015), numerical biochemical grade (Uritani et al., 1983) under UV lamp, and the metabolic and gene regulation owing to PPD. Stringent visual symptoms and scoring provide a baseline idea of PPD tolerance or susceptibility among the cassava tubers. However, understanding the ROS turnover, metabolite regulation, and key gene expression would provide insight into the PPD–resistance mechanisms. In the recent past, several genotypes were screened for PPD; however, there is always a scope to introduce new landraces to isolate PPD resistance lines and investigate precise mechanisms for alleviating cassava PPD. The present study aimed to isolate PPD–resistant lines following morphological and visual scoring and to elucidate the PPD resistance mechanisms by assessing ROS production, phenolics and antioxidant accumulations, and PPD–associated key gene expressions. The study also aimed to identify the vital metabolic compounds associated with PPD progression in resistant and susceptible cassava genotypes. The key metabolites and genes regulating PPD would be helpful for marker–assisted selection studies in cassava. Understanding the mechanisms of PPD resistance and isolation of PPD resistant lines would help augment future breeding strategies in cassava to address the challenge of PPD on storage.

## 2. Materials and methods

### 2.1. Plant growing conditions

Twelve cassava genotypes (Sree Prakash, MNP Local A, MNP Local B, H–1687, Sree Rekha, Sree Hersha, H–226, H–97, Sree Jaya, M–4, H–165, and Sree Vijaya) were grown in the Langol hill research farm, ICAR Research Complex for North Eastern Hill Region (ICAR RC NEHR), Imphal, India, located at 24°40′ N and 93°54′ E, and an elevation of 774 m masl. Ten cassava setts per genotype in three randomized replications each were planted 90 × 90 cm apart in the sandy loam soil with pH 5.4 under rainfed conditions following the recommended package of practices. The plants received 1,470–1,500 mm rainfall with 85–90% relative humidity during the crop growth period (February–December 2020). Eleven month–old tubers were harvested using a cassava harvester (CTCRI, Thiruvananthapuram, India), avoiding mechanical injury.

### 2.2. Experimental materials and storage conditions

Six well–grown cassava tubers per genotype were cleaned thrice under running tap water and used as source materials in the present study. The tubers were stored at an ambient condition in a precision plant growth chamber (Thermo Fisher Scientific, Waltham, MA, USA) in the horticulture field laboratory of ICAR RC NEHR, Imphal, India, with a day/night temperature of 20/15°C, and an average relative humidity of 79%. The occurrence of PPD was monitored morphologically, and visual scoring until 15 days of storage as recommended by Saravanan et al. (2015).

### 2.3. Morphological observations and visual scoring

The length and diameter of cassava tubers were measured by a digital vernier caliper (Aerospace, India) at 0, 3, 6, 9, 12, and 15 days of storage. The moisture content (MC) and dry matter content (DMC) were determined following the procedure of Esuma et al. (2016) with slight modification. Twenty grams of cassava tuber (without rind) from the central portion (pith region) of each genotype were collected and dried in an oven (REMI, Mumbai, India) at 105°C for 24 h to obtain a constant weight. The MC and DMC of the samples were determined following formulas 1, 2.


(1) $$\text{MC}\left(\%\right)=\frac{\mathrm{FW}-\mathrm{DW}}{\text{FW}}\times 100$$

(2) $$\text{DMC}\left(\%\right)=\frac{\text{DW}}{\text{FW}}\times 100$$

where, FW, and DW are the fresh weight and dry weight of the cassava tubers, respectively.

Visual PPD scoring was elucidated in the transverse sections (1 cm thickness) of cassava tubers cut at different portions at 15, 30, 45, 60, 75, and 90% of tuber length (proximal to distal end). Digital images of each tuber slice were captured using iPhone (Model–MNQQ2B/A, Apple, UK) at a 20 cm distance from the root section. The visual scoring was observed at 0, 3, 6, 9, 12, and 15 days by following Saravanan et al. (2015), and PPD scores between 0 and 100% were assigned to the root slices (*n* = 3) observing the physiological deterioration at the central parenchyma region (Salcedo and Siritunga, 2011). The mean PPD score for each genotype (twelve) at each time point (six) was calculated by taking the average of different scores obtained from different transverse sections (six) replicated thrice. The pattern of PPD tolerance and susceptibility among the cassava genotypes were estimated based on the minimum, and maximum PPD scores, respectively. Total PPD was calculated by averaging the scores at different time points (0–15 storage days).

### 2.4. Histochemical based PPD scoring

This study was conducted to observe the rapid accumulation of fluorescent compounds among the twelve cassava genotypes as a primary indication of PPD. The transverse sections of each tuber piece at different time points (0 to 15 days of storage) were exposed to UV Lamp (Cole–Parmer, Mumbai, India) at 365 nm frequency (Salcedo and Siritunga, 2011), and observed the bio–physiological changes in terms of fluorescence emission in each tuber slices. PPD scoring was assigned following a 0–5 point scale of Salcedo and Siritunga (2011). Score 0 denotes no symptoms under UV and naked eye; score 1 indicates bluish fluorescence under a UV–lamp but no symptoms to the naked eye; score 2 visualizes bluish fluorescence under a UV–lamp with faint bluish/browning coloration to the naked eye; score 3 depicts light browning or bluish browning with morphological deterioration; score 4 indicates moderate browning or bluish browning, and score 5 denotes severe browning or bluish browning of the tuber slices under storage.

### 2.5. Detection of ROS using 3,3 diaminobenzidine (DAB) and quantification of H_2_O_2_

Localization of reactive oxygen species in the form of hydrogen peroxide was observed following the procedure of Zidenga et al. (2012) with partial modification. DAB solution was prepared by dissolving 500 mg DAB in milli–Q water to a concentration of 1 mg mL^–1^, and the pH was adjusted to 3.8 using 2 M HCL. All the chemical reagents were procured from HiMedia (HiMedia Laboratory, Mumbai, India) and used in the present study. The transverse section of stored tuber slices (0–15 days) was incubated in 30 mL 3,3 diaminobenzidine (DAB) solution in a closed plastic container for 16–18 h under light, and the DAB solution was discarded subsequently. The tuber sections were washed thrice in distilled water and blotted with tissue paper to remove extra moisture. DAB localized the H_2_O_2_ in the presence of some haem–containing proteins and formed reddish–brown precipitation. The images were captured using iPhone (Model–MNQQ2B/A, Apple, UK), and PPD was observed based on light intensity to dark precipitation.

H_2_O_2_ was quantified following the procedures of Velikova et al. (2000). One gram of cassava root samples was homogenized in an ice bath with 5 mL trichloroacetic acid (TCA, 0.1% w/v). The homogenate was centrifuged at 8,000 rpm for 10 min, and the supernatant was collected (1 mL) and mixed with 1 mL of potassium phosphate buffer (50 mM; pH 7.0) and 2 mL of potassium iodide (1 M). The absorbance of the reaction mixture was observed at 390 nm in a spectrophotometer (Eppendorf, Hamburg, Germany), and the content of hydrogen peroxide was calculated through a standard curve derived following the formula (3).


(3) y=0.3186x-0.0287,R=20.9968

### 2.6. Estimation of total phenolics, total carotenoids, flavonoids, and anthocyanin content

Based on the morpho–histological assessment and visual scoring, two contrast cassava lines, MNP Local–A (tolerant) and Sree Prakash (susceptible), were selected for metabolites and gene expression analysis owing to progressive PPD occurrence.

#### 2.6.1. Extraction of phenolic compounds

The dried and powdered cassava tubers weighing 1 g each per replication were mixed with 10 mL of 80% ethanol and shaken well in a water bath (REMI, Mumbai, India) at 55°C for 40 min. The extract was centrifuged at 6,000 rpm for 20 min in a cooling centrifuge (Eppendorf, Hamburg, Germany). The supernatant was filtered through Whatman No. 2 filter paper, and ethanol was removed using a rotatory evaporator (Buchi, Meierseggstrasse, Switzerland) at 50°C to a final volume of 3 mL (Uarrota et al., 2014). The extracts were collected and stored at 4°C until further use.

#### 2.6.2. Total phenolic content

The total phenolic contents of the cassava extracts during PPD occurrence were determined through the Folin–Ciocalteau reagent (FCR) method with slight modification (Uarrota et al., 2014). For a 10 mL total volume, 300 μL of the extract was first mixed with 100 μL FCR reagent after adding 9.3 mL distilled water, and the contents were kept at room temperature for 10 min. Later, 300 μL Na_2_CO_3_ aqueous solution (20%, w/v) was added and incubated for 1 h. The absorbance was measured at 760 nm through a UV–visible spectrophotometer (Eppendorf, Hamburg, Germany). Total phenolic content was expressed as μg of gallic acid equivalents g^–1^ of dry extract (μg GAE g^–1^) using a standard curve (0–100 μg mL^–1^) of gallic acid.

#### 2.6.3. Total flavonoid content

Total flavonoid content was determined using the aluminum chloride colorimetric method (Uarrota and Maraschin, 2015) and standard solutions (0–100 μg/mL of quercetin in 80% methanol). For that, 0.5 mL of extract was mixed with 1.5 mL 95% ethanol (v/v), 0.1 mL 1 M potassium acetate, 0.1 mL aluminum chloride solution (10% AlCl_3_), and 2.8 mL distilled water to a total volume of 5 mL. The mixture was well mixed and incubated at room temperature for 30 min versus a blank reagent containing water instead of a sample. The absorbance was measured at 415 nm with a UV–visible spectrophotometer (Eppendorf, Hamburg, Germany). Quercetin (0, 25, 50, and 100 μg/mL) was used as the standard to quantify total flavonoids.

#### 2.6.4. Total carotenoids content

The carotenoid content was estimated following the procedure of Ceballos et al. (2012) with partial modification. Two grams of tuber samples were ground into flour and mixed with 4 mL of chilled acetone for 10 min. After homogenization, 4 mL of petroleum ether was added and mixed using an Ultra–turrax homogenizer (IKA, Wilmington, NC, USA) for 1 min. Samples were then centrifuged at 8,000 rpm for 15 min at 20°C, the supernatant was collected and mixed with 4 mL of sodium chloride (0.1 M), and again centrifuged at 8,000 rpm for 10 min. The supernatant containing the volume of dissolved extract in the petroleum ether (upper portion) was collected. The absorbance was measured at 470 nm in a UV–visible spectrophotometer (Eppendorf, Hamburg, Germany) using the absorption coefficient of β–carotene in petroleum ether (2,592 L mol cm^–1^) and calculated as represented in the equation (4).


(4) $$\left(\mu \,g\,g^{-1}\right)=\frac{A*V*10^{4}}{{\text{A}}_{1\,c\,m}^{1\%}*P}$$

where, A represents the absorbance; V, the final volume (mL); $A_{1\,c\,m}^{1\%}$ is the absorption coefficient; P is the weight of the sample in gram.

#### 2.6.5. Anthocyanin content

Anthocyanin content was determined following the methodology described by Uarrota and Maraschin (2015). Five mL methanol acidified with 1N HCl (85:15 v/v) was added to 2 g of cassava tuber flour samples, and pH was adjusted to 1.0. After spinning at 1,800 rpm for 45 min, the solution was centrifuged at 8,000 rpm for 15 min. The supernatant was collected and dried in a rotatory evaporator (Buchi, Meierseggstrasse, Switzerland) at 55°C. The dried extract was reconstituted with 2 ml of absolute methanol. The samples were diluted into two different concentrations. One with pH 1.0 using 3 M potassium chloride and the other with pH 4.5 using 3 M sodium acetate buffer. Samples were diluted five–fold to a final volume of 1 mL, and the absorbance was recorded at 520 after 30 min of incubation. The samples had no haze or sediment; thus, correction at 700 nm was omitted (Hosseinian et al., 2008). The concentration (mg/L) of each anthocyanin was calculated according to the following formula and expressed as Cy–3–glc equivalents:


(5) $$\mathrm{Anthocyanin}\,\mathrm{content}=\frac{A*\mathrm{MW}*\mathrm{DF}*10^{3}}{\varepsilon *L}$$

where A is the absorbance = (A at pH 1.0 − A at pH 4.5), MW is the molecular weight (g mol^–1^) = 449.2 g mol^–1^ for Cy–3–glc, DF is the dilution factor (0.2 ml sample is diluted to 1 ml, DF = 5), and ε is the extinction coefficient (L × cm^–1^ × mol^–1^) = 26,900 for Cy–3–glc, where L (path length in cm) = 1.14.

### 2.7. Compound analysis using GC–MS

We have performed Gas chromatography–mass spectrometry (GC–MS), to elucidate the major compounds associated with PPD in two promising cassava tubers before (0 day) and after the onset of PPD (72 h). Dried cassava powders (150 mg) were dissolved in 30 ml of n–hexane (GCMS grade, Sigma Aldrich, Bengaluru, India) for 72 h. The solvent was evaporated and concentrated using a rotary vacuum evaporator (Buchi, Meierseggstrasse, Switzerland). The concentrated solvent extract was syringe filtered through 0.45, and 0.5 μL sample was injected in the GC–MS (Thermo Scientific, Waltham, MA, USA) following the cold maceration method (Devi et al., 2021). The compounds were identified based on the reverse search index (RSI 800–1,000) through NIST library matching. Gas chromatography–mass spectrometry analysis was employed using a Trace 1300 series (GC) interfaced with a mass spectrometry (TSQ duo) fitted with a TG–5MS fused silica capillary column (30 m × 0.25 mm; 0.25–μm film thickness). For GC, the oven temperature range was programmed from 40 to 280°C at 5°C min^–1^, and helium was used as a carrier gas at a flow rate of 1.0 mL min^–1^ for analysis. For the mass detector, the mass transfer line and the ion source temperature were set at 280 and 240° C, respectively.

### 2.8. Gene expression analysis

Expression of PPD–associated genes (*APX2, APX3, PAL*, and *AP*) were analyzed in two contrast cassava lines, MNP Local–A (tolerant), and Sree Prakash (susceptible) during progressive PPD occurrence at 0, 3, 6, 9, 12, and 15 days following quantitative real–time polymerase chain reaction (qRT–PCR). Total RNA was isolated from the cassava tuber slices using the GSure Plant RNA Kit (GCC Biotech (I) Pvt. Ltd., Kolkata, India) following the manufacturer’s protocol. The quantity and quality of the isolated RNA were determined using NanoDrop (Thermo Scientific, Waltham, MA, USA). RNA samples with 260/280 ratio of 1.9–2.1 were used for subsequent expression analysis. RNA integrity was visualized in 1.2% agarose gel electrophoresis with two clear bands of 28S/18S ribosomal RNA. cDNA was synthesized from the isolated RNA using dual step RT–PCR kit (GCC Biotech (I) Pvt. Ltd., Kolkata, India) and processed for PPD–associated gene expression analysis.

The expression of four PPD–associated genes, *APX2, APX3, PAL*, and *AP*, were successfully amplified with forward and reverse primer sequences (Table 1). The optimization and standardization of primers used in real–time PCR were done using a gradient PCR assay wherein the 18S rRNA was used as an internal control (Iyer et al., 2010). After the standardization, the primers were used in the real–time quantitative PCR assay. PCR was performed using EmeraldAmp GT PCR Master Mix (2X Premix, TaKaRa, Japan) with gene–specific primers. For a total volume of 10 μl, 1 μl template (cDNA), 5 μl PCR 2X MM and 0.5 μl forward primer and 0.5 μl reverse primer, and 3 μl nuclease–free molecular grade water was mixed and ran with an initial denaturation at 94°C for 2 min was followed by 30 cycles of 94°C for 30 s, 50°C/55°C for 30 s, and 72°C for 45 s. The final extension was accomplished at 72°C for 5 min. As a loading control, 18S rRNA was used as a reference. After PCR, the primers/genes were validated for specific amplification through 1.5% agarose gel electrophoresis.

**TABLE 1:** List of primers selected for relative gene expressions of *APX2*, *APX3*, *PAL*, and *AP* genes in the tuber slices of the two contrast cassava genotypes under the progressive postharvest physiological deterioration (PPD).

Primers	Primer sequences (5′–3′)	Sequence length	GC (%)	Tm (°C)	Amplicon (bp)
*APX2*–F	TTCCTTCTTCCAGGTGCTCTTG	22	50.00	60.25	344
*APX2*–R	CCGACCATCATCACATTCAA	20	45.00	55.25	
*APX3*–F	AGGTGCTGATCACTTGAGAGAG	22	50.00	60.25	500
*APX3*–R	CCGACCATCATCACATTCAA	20	45.00	55.25	
*PAL*–F	GCTGCTGCTATTATGGAACA	20	45.00	55.25	400
*PAL*–R	CTAGGATTCCTTCCTCCTGT	20	50.00	57.30	
*AP*–F	CAAGCAGAATGCAACACTGGAG	22	50.00	60.25	450
*AP*–R	CACGAGGTTTAGTTCAAGCC	20	50.00	57.30	
*18S*–F	CAGACTGTGAAACTGCGAATGG	22	50.00	60.25	520
*18S*–R	ATTGGAGCTGGAATTACCGC	20	50.00	57.30	

The qRT PCR was carried out using KAPA SYBR^®^ FAST Universal (KAPA BIOSYSTEMS) on QuantStudio^T*M*^ 5 System (Thermo Fisher Scientific, Waltham, MA, USA) in a 96–well plate format according to the manufacturer’s instructions. The total volume of each reaction was 10 μl comprising of 3.4 μl nuclease–free water, 5 μl SYBR^®^ FAST qPCR Master mix 2X (Applied Biosystems, Waltham, CA, USA), 0.2 μl forward primer (10 μM), 0.2 μl reverse primer (10 μM) and 0.2 μL ROX Low (50X) and 1 μl (50 ng) of cDNA template. A single melting peak in the dissociation curves confirmed primer specificity. 18s rRNA gene was used as the internal reference gene.

The mixture was dispensed into Step One™ Real–Time PCR system (Applied Biosystems, Waltham, CA, USA) with a reaction plate of 96 wells. The qRT–PCR plates were sealed using adhesive PCR seals to protect against evaporation and dispersion. The qRT–PCR amplification program was set at 94°C for 2 min followed by 39 cycles of 94°C for 15 s, 60°C for 1 min, and a melting curve analysis was performed at 95°C for 15 s, 60°C for 1 min and 95°C for 1 s. The dissociation temperature range extends from 65 to 95°C. The ΔΔ Ct method was used to find the relative gene expression by taking the 18s rRNA internal control gene for data normalization. The relative expression level was analyzed using the Livak (ΔΔCT) method (Livak and Schmittgen, 2001), where ΔΔCt = (Ct target—Ct 18s rRNA) time x-(Ct target—18s rRNA) time 0. For confirmation, the qRT–PCR products were run on 1.5% agarose gel with 1X TBE buffer (w/v) at 100 V for 1 h. A 100 bp DNA ladder (New England Biolabs, UK) was used to confirm that the product sizes match those of the selected genes. The relative expression of up and down–regulated genes associated with ROS turnover, Cell wall degrading, PCD and signaling pathways, and cyanogenic glucoside biosynthesis regulations were studied.

### 2.9. Statistical analysis

Statistical analyses were carried out using IBM SPSS Statistics 26 following one–way analysis of variance (ANOVA) in a completely randomized design (CRD). The standard deviations were estimated following the formula, σ = Σ(Xi-μ)2 N-1 at *P ≤ 0.05*, where σ is the standard deviation, Xi is the value from the population, μ is the population mean, and N is the population size. Duncan’s multiple range test (DMRT) was performed at *P ≤ 0.05* to evaluate the significant differences in PPD. The relationship between physiological and biochemical scores, ROS turnover, metabolite accumulation, and gene expression was assessed using linear regression by taking the mean score of each genotype (*n* = 36 at each time point).

## 3. Results

### 3.1. Morphological observations

The average length and diameter of the cassava tubers selected for the PPD study are presented in Table 2. The mean tuber length and diameter varied from 38.12 to 58.76 cm and 3.01 to 4.71 cm, respectively. The mean moisture content (MC) of cassava tubers ranged from 56.2 (H–165) to 64% (Sree Prakash). On the contrary, the dry matter content (DMC) ranged between 36.01 (Sree Prakash) and 42.80% (H–165).

**TABLE 2:** Length (cm), diameter (cm), moisture content (MC, %), and dry matter content (DMC, %) of the tubers of twelve cassava genotypes.

S.No.	Varieties	Length (cm)	Diameter (cm)	MC (%)	DMC (%)
1	Sree Prakash	44.86 ± 13.29^ab^	4.71 ± 1.16^d^	64.00 ± 2.68^f^	36.01 ± 2.68^a^
2	MNP Local A	39.30 ± 13.15^a^	3.34 ± 0.41^ab^	59.77 ± 1.23^bcd^	41.23 ± 0.85^bc^
3	MNP Local B	38.48 ± 12.19^a^	3.32 ± 0.52^ab^	56.86 ± 3.18^ab^	40.80 ± 1.4^abc^
4	H–1687	43.94 ± 12.68^ab^	4.13 ± 0.76^bcd^	58.29 ± 1.39^ab^	42.71 ± 3.09^c^
5	Sree Rekha	39.22 ± 10.86^a^	3.91 ± 0.43^abcd^	61.71 ± 1.09^def^	38.29 ± 1.09^abc^
6	Sree Hersha	58.76 ± 5.03^b^	4.08 ± 0.74^bcd^	63.32 ± 0.53^ef^	36.68 ± 0.53^ab^
7	H–226	46.44 ± 11.61^ab^	4.19 ± 0.78^bcd^	61.43 ± 0.25^cdef^	41.57 ± 5.25^bc^
8	H–97	39.82 ± 18.22^a^	3.01 ± 0.66^a^	58.36 ± 0.39^ab^	41.64 ± 0.39^bc^
9	Sree Jaya	41.08 ± 4.67^ab^	3.52 ± 0.81^abc^	60.33 ± 1.03^bcd^	42.01 ± 5.07^c^
10	M–4	43.86 ± 3.89^ab^	4.50 ± 0.25^cd^	60.85 ± 1.37^bcde^	39.82 ± 2.44^abc^
11	H–165	38.26 ± 16.34^a^	3.83 ± 0.35^abcd^	56.20 ± 1.47^a^	42.80 ± 0.61^c^
12	Sree Vijaya	38.12 ± 15.46^a^	3.79 ± 0.89^abcd^	58.72 ± 0.32^abc^	41.28 ± 0.32^bc^

Values are the mean of three replications ± the standard deviation (SD) at *P* ≤ 0.05. The same superscript alphabets in the same column are not significantly different according to Duncan’s multiple range test (DMRT) at *p* ≤ 0.05.

### 3.2. Visual PPD scoring

**FIGURE 1:**
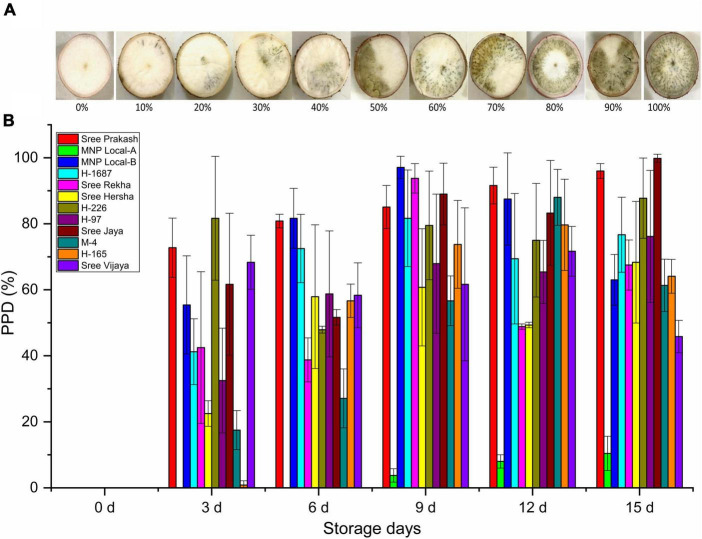
Postharvest Physiological Deterioration (PPD,%) observed by subjective visual scoring in the tuber slices of twelve cassava genotypes at 0, 3, 6, 9, 12, and 15 days of storage **(A)**, and the scale used for visual scoring **(B)**. Values are the mean of three replications, and the bars represent the standard deviation at *P* ≤ 0.05.

Figure 1 depicted the PPD observed by subjective visual scoring in the tuber slices of twelve cassava genotypes at 0, 3, 6, 9, 12, and 15 days of storage. The PPD was evaluated on the central parenchyma surface of the transverse sections based on the spreading pattern or portion of vascular streaking to the neighboring parenchyma tissues, not on the intensity of discoloration. The average PPD score was significantly higher (71%) in Sree Prakash and lowered in MNP Local A (9%). At 15 days of storage, MNP Local A exhibited the lowest PPD score of 10% compared to other tested tubers.

### 3.3. Histochemical based PPD scoring

**FIGURE 2:**
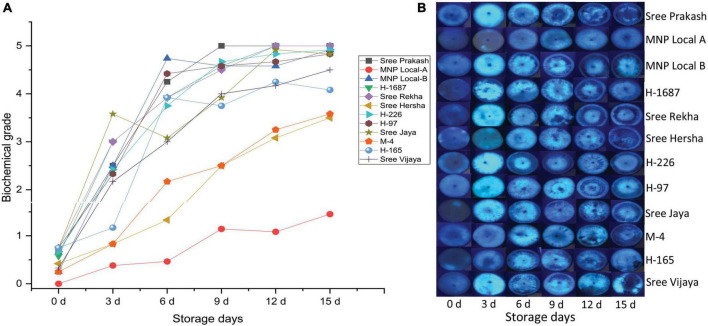
Postharvest Physiological Deterioration (PPD) scores **(A)** based on physiological and biochemical changes in the tuber slices of twelve cassava genotypes at 0, 3, 6, 9, 12, and 15 days of storage observed under ultraviolet (UV) light **(B)**. Values are the mean of three replications, and the bars represent the standard deviation at *P* ≤ 0.05.

The transverse section of each tuber slice at 0, 3, 6, 9, 12, and 15 days of storage was exposed to UV light, and the fluorescence images depicting the physiological and biochemical changes were presented in Figure 2. Accumulation of fluorescence compounds was gradually prominent after the storage period, leading to PPD. The biochemical grading was determined based on changes under UV light (Figure 2A). MNP Local A registered the consistently lower biochemical grade at 0, 3, 6, 9, 12, and 15 days of storage compared to other tested genotypes. On the other hand, Sree Prakash, Sree Rekha, H–226, and H–97 exhibited rapid accumulation of the fluorescent compounds (Figure 2A).

### 3.4. Detection of ROS using 3,3 diaminobenzidine (DAB) and quantification of H_2_O_2_

**FIGURE 3:**
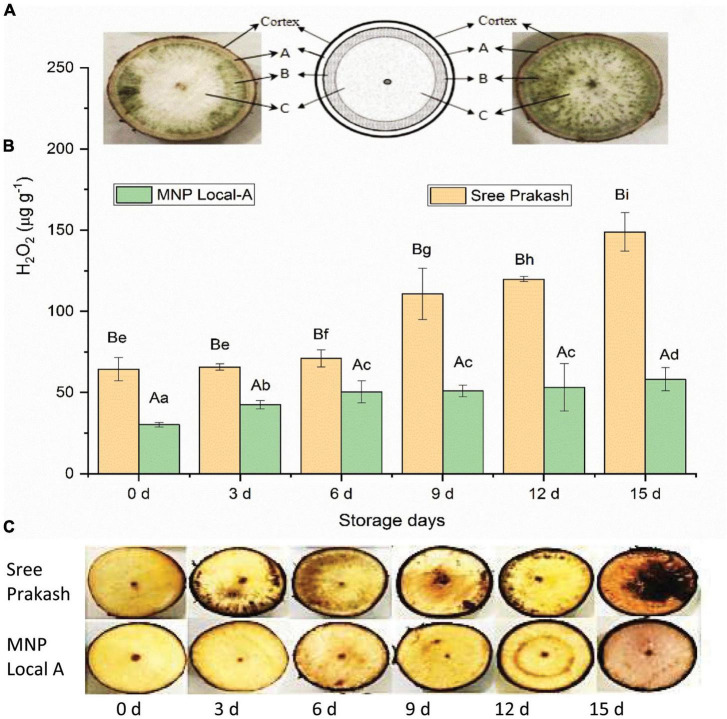
Occurrence of reactive oxygen species (ROS) in terms of H_2_O_2_ in the tuber slices of the two contrast cassava genotypes, Sree Prakash, and MNP Local A, under the progressive occurrence of postharvest physiological deterioration (PPD) at 0, 3, 6, 9, 12, and 15 days of storage. **(A)**: outermost parenchyma tissue; **(B)**: intervening tissue; and **(C)**: innermost starchy tissue. Values are the mean of three replications, and the bars represent the standard deviation at *P* ≤ 0.05. Different letters in uppercase represent significant differences between the two genotypes, and lowercase represents significant differences among the storage days in each genotype according to Duncan’s Multiple Range Test (DMRT).

The illustrative diagram of the two contrast cassava genotypes, Sree Prakash and MNP Local A, showed differential ROS zones in tuber cross–sections (Figure 3A). Figure 3B depicts reactive oxygen species (ROS) in terms of H_2_O_2_ in the tuber slices under the progressive occurrence of postharvest physiological deterioration (PPD) at 0, 3, 6, 9, 12, and 15 days of storage.

H_2_O_2_ content significantly increased with progressive storage periods at 0, 3, 6, 9, 12, and 15 days (Figure 3B). The average H_2_O_2_ content of Sree Prakash was increased from 64 μg g^–1^ (0 day) to 141 μg g^–1^ (15 day), whereas MNP Local A exhibited lower H_2_O_2_ content in the range of 29 μg g^–1^ (0 day) to 57 μg g^–1^ (15 day).

Localization of H_2_O_2_ in different cassava tubers at 0, 3, 6, 9, 12, and 15 days was presented in Figure 3C. The reddish–brown precipitation appeared after 3 days of storage except in MNP Local A and Sree Hersha tubers. Overall, less reddish/brown precipitation was observed in MNP Local A at each time point, indicating a lower accumulation of H_2_O_2_.

### 3.5. Estimation of total phenolics, total carotenoids, flavonoids, and anthocyanin content

**FIGURE 4:**
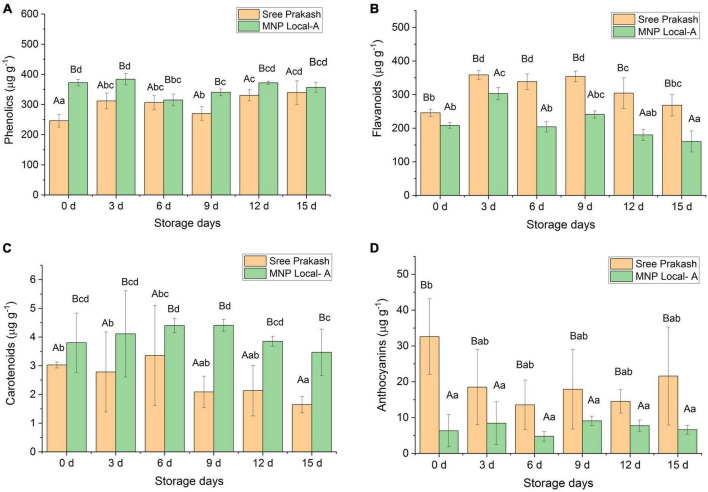
Changes in total phenolics **(A)**, flavonoids **(B)**, carotenoids **(C)**, and anthocyanins **(D)** in the tuber slices of the two contrast cassava genotypes, Sree Prakash, and MNP Local A under the progressive occurrence of postharvest physiological deterioration (PPD) at 0, 3, 6, 9, 12, and 15 days of storage. Values are the mean of three replications, and the bars represent the standard deviation at *P* ≤ 0.05. Different letters in uppercase represent significant differences between the two genotypes, and lowercase represents significant differences among the storage days in each genotype according to Duncan’s Multiple Range Test (DMRT).

Phenolics have a significant influence on PPD inhibition. The total phenolics content increased significantly with the storage time at 0, 3, 6, 9, 12, and 15 days (Figure 4A). The phenolic content of Sree Prakash and MNP Local A ranged from 245 μg g^–1^ to 339 μg g^–1^ and 314 to 372 μg g^–1^, respectively (Figure 4A). MNP Local A exhibited higher phenolics across the storage time when compared with Sree Prakash.

Flavonoids contents were recorded as 245 to 358 μg g^–1^ and 160 to 303 μg g^–1^ in Sree Prakash and MNP Local A, respectively (Figure 4B). Sree Prakash showed higher flavonoids than in MNP Local A, which appeared to be increased at 3 days of storage and further decreased with PPD progression.

Figure 4C depicts the carotenoid contents in MNP Local A and Sree Prakash at 0, 3, 6, 9, 12, and 15 days of storage. MNP Local A (3.47–4.41 μg g^–1^) possessed higher carotenoids than Sree Prakash (1.64–3.36 μg g^–1^). Overall, the carotenoid content decreased with increased storage time. The rate of decrease in carotenoids was higher in Sree Prakash than in MNP Local A.

The anthocyanin content was observed to be significantly higher in Sree Prakash, which was in the range of 13.56–32.60 μg g^–1^. The anthocyanin content was as low as 1.24–5.97 μg g^–1^ in MNP Local A (Figure 4D).

We have found a positive correlation of hydrogen peroxide (*y* = 1.01x–31.53, *R*^2^ = 0.71), flavonoids (*y* = 0.40x–67.74, *R*^2^ = 0.45), and anthocyanins (*y* = 1.69x ± 17.22, *R*^2^ = 0.11). However, PPD was negatively correlated with total phenolic (*y* = –0.33x ± 150.62, *R*^2^ = 0.11) and carotenoids (*y* = –35.59x ± 155.98, *R*^2^ = 0.65).

### 3.6. Compound analysis using GC–MS

**FIGURE 5:**
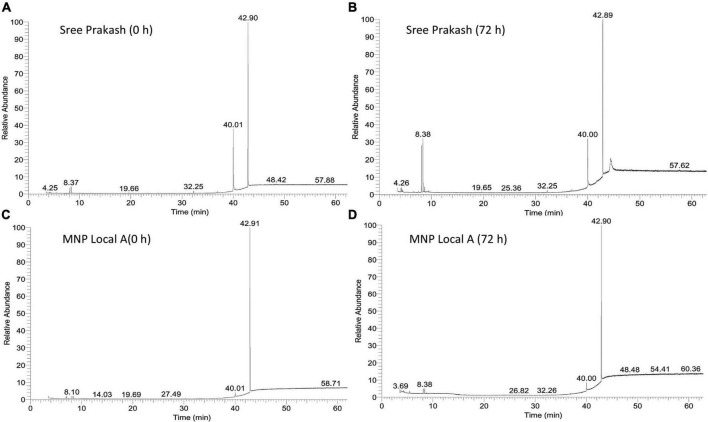
Representative total ion chromatogram of cassava tuber extracts, Sree Prakash **(A,B)**, and MNP Local A **(C,D)** at 0, and 72 h of postharvest physiological deterioration (PPD) occurrence by using Gas chromatography–mass spectrometry (GC–MS).

**FIGURE 6:**
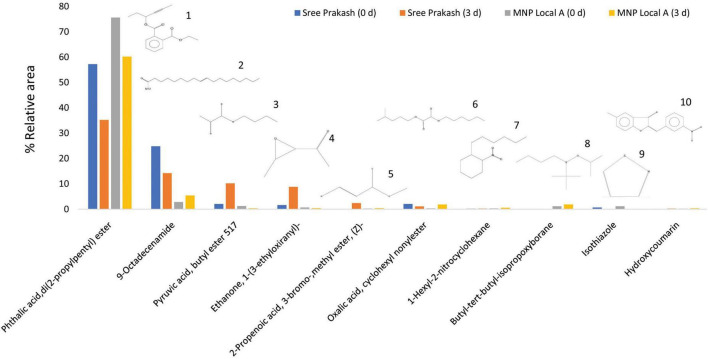
Top ten compound constituents in two cassava tuber extracts, Sree Prakash, and MNP Local A at 0, and 72 h of postharvest physiological deterioration (PPD) occurrence by using solid phase micro extraction–gas chromatography–mass spectrometry (GC–MS). 1. Phthalic acid, di(2–propylpentyl) ester; 2. 9–Octadecenamide; 3. Pyruvic acid, butyl ester 517; 4. Ethanone, 1–(3–ethyloxiranyl)–; 5. 2–Propenoic acid, 3–bromo–,methyl ester, (Z)–; 6. Oxalic acid, cyclohexyl nonylester; 7. 1–Hexyl–2–nitrocyclohexane; 8. Butyl–tert–butyl–isopropoxyborane; 9. Isothiazole; 10. 4,6–Dimethyl–3′–hydroxy–2–benzylidene–coumaran–3–one (Hydroxycoumarin).

Figure 5 depicts the representative total ion chromatogram of cassava tuber extracts, Sree Prakash, and MNP Local A at 0 and 72 h of PPD occurrence using Gas chromatography–mass spectrometry (GC–MS). The GC–MS result in our study revealed that the primary compound, Phthalic acid, di(2–propylpentyl) ester (MW–390), was identified with a retention time of 42.91, which was varied among the tested genotypes, Sree Prakash (57.21 and 35.21%), and MNP Local A (75.58 and 60.21%) at 0, and 72 h of PPD, respectively. The major compounds identified in the two promising cassava tubers before and after the onset of PPD have been presented in Figure 6. In the resistant line MNP Local A, all the metabolic compounds increased under PPD, whereas 9–Octadecenamide, Oxalic acid, cyclohexyl nonyl ester, 1–Hexyl–2–nitrocyclohexane, Butyl–tert–butyl–isopropoxyborane, and 4,6–Dimethyl–3′–hydroxy–2–benzylidene–coumaran–3–one (Hydroxycoumarin) increased remarkably, which indicated their close association with PPD inhibition mechanisms. On the other hand, Pyruvic acid, butyl ester 517, Ethanone, 1–(3–ethyloxiranyl) –, 2–Propenoic acid, and 3–bromo–, methyl ester, (Z)–increased highly in susceptible, Sree Prakash (Figure 6). Oxalic acid is a vital antinutrient factor associated with resistance mechanisms. In our study, oxalic acid accumulation was decreased in susceptible Sree Prakash and increased in tolerant MNP Local A. Similarly, high occurrence of Pyruvic acid, butyl ester 517, and Ethanone, 1–(3–ethyloxiranyl)– reasoned for susceptibility in Sree Prakash, which was decreased in MNP Local A.

### 3.7. Gene expression analysis

**FIGURE 7:**
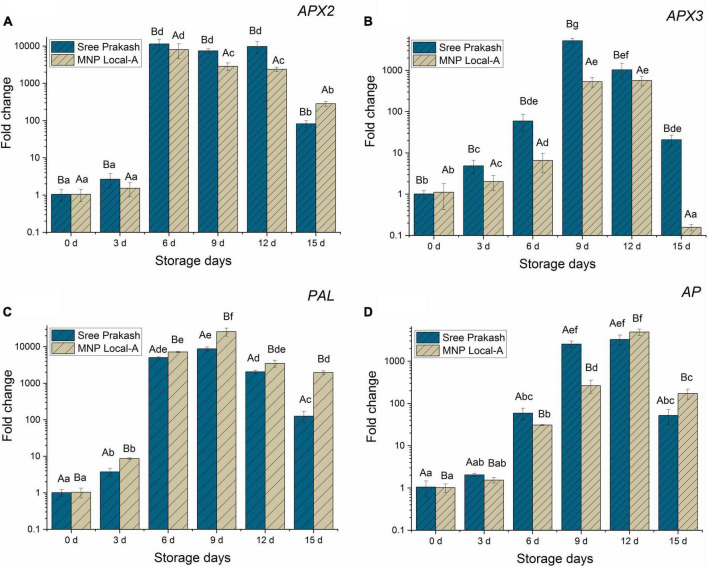
Relative gene expressions of *APX2* **(A)**, *APX3* **(B)**, *PAL* **(C)**, and *AP* **(D)** genes (fold change in Log_10_) in the tuber slices of the two contrast cassava genotypes, Sree Prakash, and MNP Local A under progressive occurrence of postharvest physiological deterioration (PPD) at 0, 3, 6, 9, 12, and 15 days of storage. Values are the mean of three replications, and the bars represent the standard deviation at *P ≤ 0*.05. Different letters in uppercase represent significant differences between the two genotypes, and lowercase represents significant differences among the storage days in each genotype according to Duncan’s Multiple Range Test (DMRT).

The expression of four cassava genes, *APX2, APX3, PAL*, and *AP*, associated with PPD occurrence in Sree Prakash and MNP Local A at different storage durations were investigated concerning the *18S rRNA* gene (Figure 7). The cycle threshold (CT) values of *APX3, APX2, PAL, AP*, and the reference *18S rRNA* genes were estimated, and the relative expressions were analyzed using the ΔΔCT method (Livak and Schmittgen, 2001).

Figure 7A depicted a rapid escalation in the *APX2* gene at 6 days, which continued to up–regulated until 12 days and declined at 15 days of storage. Expression of the *APX2* gene was 10–fold higher than *APX3, PAL*, and *AP* gene expression under PPD occurrence at 15 days.

Ascorbate peroxidase3 (*APX3*) was firmly up–regulated in the cassava tubers until 9–12 days, which was subsequently declined at 15 days of storage (Figure 7B). The expression of the *APX3* gene was higher in Sree Prakash than in MNP Local A, which indicated its association with the overproduction of ROS (H_2_O_2_). Sree Prakash tubers exhibited higher H_2_O_2_ as oxidative burst to encounter PPD.

The expression of the *PAL* gene at different time points of PPD has been presented in Figure 7C. Our study revealed that the *PAL* gene was expressed consistently higher at 6–15 days of PPD, which indicated the association of *PAL* in the late event of PPD. Expression of the *PAL* gene was comparatively higher in MNP Local A at each stage of PPD compared with Sree Prakash, which indicated the defense ability against PPD.

Expression of *AP* gene activity increased significantly during 6–12 days and declined at 15 days of PPD. The *AP* gene expression was higher in MNP Local A at the late stage at 12 and 15 days compared to Sree Prakash.

## 4. Discussion

Our study revealed significant morphological and physiological variations among the cassava tubers. Sree Prakash exhibited higher moisture and lower dry matter content, whereas MNP Local A showed lower moisture and higher dry matter content. Previous reports suggested that cassava tubers containing higher moisture and low dry matter content led to significantly higher PPD (Uarrota et al., 2016). The DMC values of 31.45–40.74% are considered potential economic values for cassava products (Teye et al., 2011). The aggregation of DMC within the tuber decreases from the proximal to the distal end, and the middle tuber portion illustrates the tuber’s representative DMC values (Teye et al., 2011). Therefore, the middle portion of the tuber has been selected in the present study for the PPD scoring and mechanism assay. Extending the shelf life of cassava tubers to a minimum of 2 weeks would be a significant outcome in cassava utilization which resolves 90% of the deterioration constraints for its commercialization (Oirschot et al., 2000).

Vascular streaking or vascular discoloration, which depicts the primary or physiological degradation, is the general term for the formation of blackish or brownish radial veins or streaks close to the xylem vessels in the tuber pith tissues. This symptom prominently appeared in most cassava genotypes within 2–3 days after harvest except in MNP Local A, Sree Hersha, M–4, and H–165 genotypes. Afterward, Sree Hersha, M–4, and H–165 exhibited PPD symptoms after 6 days of storage. However, MNP Local A showed mere vascular striking after 12 days of storage.

Postharvest physiological deterioration results in quick accumulation of fluorescent chemicals under UV light, which also triggers the onset of physiological and metabolic damage (Buschmann et al., 2000). The fluorescent compound includes hydroxycoumarin, such as scopoletin, esculin, and scopolin. Among this hydroxycoumarin, scopoletin is the most prominent fluorescent compound, which has shallow occurrence in fresh tubers and gradually increases upon periodic storage (Rickard and Coursey, 1981). In our study, a sudden increase in fluorescent compounds after 3 days of storage was observed in all the genotypes except in MNP Local A and Sree Hersha, which indicated the occurrence of hydroxycoumarin and scopoletin in particular. At 6 and 9 days of storage, there is a decline of fluorescent compounds, which indicates the association of scopoletin, followed by a slight increase in scopolin and esculin, as reported by Buschmann et al. (2000). After 9 days of storage, there is a reduction in fluorescent compounds, which indicates complete depletion of hydroxycoumarin. UV visualization of the fluorescent compound is an early event that could use for stringent PPD scoring.

The cassava root transverse section consists of three main parts. Part A: the outermost parenchyma tissue; part B: the intervening tissue; and part C: the innermost starchy tissue. The vascular streaking was initiated in part B, which also exhibited higher fluorescence and phenolic compounds compared to parts A and C (Safafar et al., 2015). Most of the cassava tubers produced blackish–brown discoloration and pigmentation throughout the root surface. However, Sree Prakash tubers showed vascular streaking in the form of a ring around the inner pith region (Figure 3). The physiological vascular striking around the pith was reasoned due to the ROS accumulation and microbial activity during PPD (Taniguchi et al., 1984). Most cassava roots showed rings and spreading patterns of vascular streaking due to the overproduction of reactive oxygen species (ROS) such as H_2_O_2_.

Reactive oxygen species (ROS) increased at early PPD stages, which indicated the role of ROS in the form of H_2_O_2_, hydroxyl radical (HO), superoxide anion radical (O^–2^), and singlet oxygen (O–O) associated with PPD (Reilly et al., 2004). Upon PPD, a rapid oxidative burst occurred due to over production of hydrogen peroxide and superoxide, which were inversely correlated with phenolic compounds (Buschmann et al., 2000).

The DAB localized H_2_O_2_ in the presence of haem–containing protein to form a reddish–brown precipitate (Sahoo et al., 2020). Cassava tuber sections at PPD events showed more dark precipitation than the fresh tubers due to a higher occurrence of H_2_O_2_. However, a mechanical injury may cause an oxidative burst with blackish precipitation induced by cyanogenesis and H_2_O_2_ accumulation (Zidenga et al., 2012). Cyanogenesis further inhibits mitochondrial respiration, resulting in a burst of ROS–led PPD.

The amount of phenolics in plants has a significant role in their overall antioxidant activity, as reported by numerous researchers (Negro et al., 2003; Li et al., 2009). Phenolic compounds are well–derived antioxidants that effectively manage free radicals produced owing to PPD (Zeb, 2020). The antioxidant properties of phenolic content are often achieved by neutralizing lipid free radicals that contain oxygen (ROS) and halting the conversion of hydroperoxides into free radicals. Cassava tuber tissues accumulate phenolic compounds, such as scopolin, scopoletin, and diterpenoids, to combat the negative consequences of ROS that occur during PPD (Yi et al., 2011). The increase in total phenolics was higher in MNP Local A, which effectively inhibited the occurrence of PPD compared to Sree Prakash. Phenolics were significantly and negatively correlated with PPD severity (Blagbrough et al., 2010). Mechanism of phenolic compounds occurred through hydrogen atom transfer (HAT), single electron/proton transfer (SET/SPT), sequential transfer of proton, and transition metal chelation (TMC) with an ultimate goal of catalyzing H_2_O_2_ into O–O.

Flavonoids minimize stress–associated ROS homeostasis, thereby inducing the phytoanticipins, the constructive defense mechanism against PPD (Uarrota and Maraschin, 2015). Buschmann et al. (2000) reported accumulation of flavonoid at early stages of 4–6 days storage which depletes after 9 days. In our study, flavonoids increased in the cassava tubers during the first 3 days of storage, which decreased at 6, 9, 12, and 15 days in the case of MNP Local A, whereas flavonoids increased up to 9 days in Sree Prakash and further decreased at 12, and 15 days.

Higher carotenoid content indicates low PPD in cassava tubers (Morante et al., 2010). In our study, the total carotenoid content significantly increased with a progressive storage period in MNP Local A. However, carotenoids in Sree Prakash increased until 6 days and rapidly decreased at 9, 12, and 15 days (Figure 4C). Enhanced carotenoids are vital in delaying PPD in cassava by inhibiting cellular oxidative damage (Liu et al., 2019). PPD expression and β–carotenoid content were influenced by additive gene actions, whereas a large panel of volatile and non–volatile metabolites were governed by non–additive gene action (Athanase and Rob, 2019). Accumulation of stress–related metabolites is a significant indicator of PPD inhibition in the presence of a wide range of the compounds, such as phytosterols, diterpenes, and fatty acid derivatives, which subsequently induce hydroxycoumarin as visualized using UV lights in the present study. In our study, Phthalic acid, di(2–propylpentyl) ester, was identified as one of the primary compounds in both the cassava tubers under PPD progression. Phthalic acid, di (2–propylpentyl) ester present in raw cassava tubers as waste accumulation and distributed in the root tissues, and the proportion of apoplastic and symplastic movement in the cassava roots depends on the percentage composition (Penido et al., 2019). Phthalic acid is a plasticizer and toxic compound, which is reduced upon boiling before consumption. Anthocyanins are water–soluble, colored pigments that belong to the polyphenolic compounds in a glycosylated form that play a significant role in PPD inhibition (Uarrota and Maraschin, 2015). In our study, fresh tubers of Sree Prakash contained higher anthocyanin, which rapidly depleted upon the occurrence of PPD. However, anthocyanin had a non–significant effect on MNP Local A at different time points (Figure 4D), which was a good indicator of PPD inhibition. PPD was positively related to anthocyanins (*y* = 1.69x ± 17.22, *R*^2^ = 0.11). PPD progression is significantly correlated with anthocyanin and flavonoids; however, inversely related to phenolics and carotenoids (Zainuddin et al., 2018).

Our results indicated that the complexity of the ROS and physiological and biochemical changes occurring in cassava tubers are more strongly related to PPD than the post–harvest wounding; upon PPD occurrence, a set of biochemical events takes place in a closely orchestrated cascade of polyphenols to repair the tissue damage caused due to ROS overproduction.

The most crucial peroxidase in the detoxification of H_2_O_2_ is ascorbate peroxidase (*APX*), which uses ascorbate’s reducing ability to catalyze the conversion of H_2_O_2_ to water (Negro et al., 2003). Rapid induction of ascorbate and activation of peroxidases could scavenge the overproduction of H_2_O_2_ to form H_2_O and O_2_^–^ during PPD (Reilly et al., 2004; Xu et al., 2013). However, our study noticed no significant expression in *APX2* at 0 and 3 days of storage. Vanderschuren et al. (2014) reported that *APX2* occurred late during PPD progressions, and *APX* abundance does not comply with ROS turnover at early PPD progression (Owiti et al., 2011).

Furthermore, up–regulated *APX3* gene mechanistically impairs the negative consequences of the ROS that occur during PPD (Reilly et al., 2007; Uarrota et al., 2016). ROS–regulating gene expression typically increases oxidative stress resistance vis–à–vis enhanced ROS scavenging machinery, thereby delaying PPD (Reilly et al., 2007). *APX3* also has a stringent role in the induction of non–enzymatic ascorbic acid (AsA) and glutathione (GSH), associated with the ascorbate cycle to scavenge ROS (H_2_O_2_) and modulate PPD (Vanderschuren et al., 2014).

Phenylalanine ammonia–lyase is one of the vital enzymes to the core reactions of general phenylpropanoid metabolism and controls the ROS flux through several pathways (Bennett and Wallsgrove, 1994). As the key entry point into phenylpropanoid metabolism, altered *PAL* expression could affect diverse pathways, including the synthesis of wound healing components such as lignin and suberin and signaling compounds such as salicylic acid (Reilly et al., 2003). In our study, Expression of the *PAL* gene was comparatively higher in MNP Local A at each stage of PPD compared with Sree Prakash.

In the present study, MNP Local A exhibited overall gene expression, indicative of better ROS and PPD management strategies than Sree Prakash. *AP* gene played a potential role in late events of programmed cell death (Iyer et al., 2010) and protein degradation (Lopez et al., 2005). *AP* genes are also designated as wound–impairing genes regulated under PPD progression in cassava which also regulates ROS production and phenolic compounds (Fernando et al., 2002). Aspartic protease, a class of proteolytic enzymes, catalysis the peptide substrates in the presence of one or more aspartate–bound activated water molecules. The *PAL* and *AP* genes, which are highly expressed in PPD–resistant MNP Local A, could be used for marker–assisted selection and future cassava breeding programs.

## 5. Conclusion

Our study established the interaction between PPD–ROS turnover regulated by the key *APX2* and *APX3* genes, fine–tuned with phenolic metabolites biosynthesis and wound healing components expressed by *PAL* and *AP* genes, to modulate PPD in cassava. The visual score indicated severe PPD at 12–15 days. However, MNP Local A exhibited the lowest score of 10% even after 15 days of PPD, which was a good indication of PPD resistance. The major compound, Phthalic acid, di(2–propylpentyl) ester, was identified in both the cassava tubers under PPD progression. Visualization of fluorescence compound hydroxycoumarin (4,6–Dimethyl–3′–hydroxy–2–benzylidene–coumaran–3–one) was detected as an early event at 3–6 days which further declined at 9–15 days and indicated depletion of scopoletin. Overproduction of H_2_O_2_ reasoned for a ring and spreading type of vascular streaking, which was localized using DAB at an early stage of 3 days, which could be used for PPD screening at an early stage. The accumulated H_2_O_2_ coupled with *APX2* and *APX3* genes were significantly expressed at 3–12 days and declined at 15 days. The biosynthesis of phenolic compounds, flavonoids, carotenoids, and anthocyanin is inversely correlated with H_2_O_2_ accumulation, which acts as a ROS inhibitor and PPD modulator. MNP Local A showed a low PPD score coupled with low ROS turnover, higher metabolite accumulation, and gene expression. The early events components, such as H_2_O_2_ and the phenolics antioxidants, could be used for rapid and large–scale screening of cassava genotypes for PPD resistance. MNP Local A could be used as a potent genetic resource for the PPD–targeted cassava improvement program. The PPD–resistant genotype(s) may be popularized for large–scale production, consumer acceptability, and better marketability. The result of the study also provides insight into the genetic engineering of cassava involving *APX* and *PAL* genes to regulate ROS turnover and phenylpropanoid biosynthesis to overcome PPD.

## Data availability statement

The original contributions presented in this study are included in this article/supplementary material, further inquiries can be directed to the corresponding author.

## Author contributions

EW and MS: conceptualization. EW, SS, YR, and MS: data curation. EW, AM, NS, SS, and MS: formal analysis. EW, AM, and NS: investigation. EW, MD, SS, and MS: methodology. MS, CD, SS, and SR: resources. EW, SL, TC, AN, IB, YD, and LS: software. EW, ST, CD, and CK: writing – original draft. MS, MD, YR, SM, and SD: writing – review and editing. All authors read and agreed to the published version of the manuscript.
